# Exploring Healthcare Provider Experiences with the EXCEL Exercise Referral Pathway for Individuals Living with and Beyond Cancer

**DOI:** 10.3390/curroncol32030181

**Published:** 2025-03-20

**Authors:** Alexandra Finless, Mannat Bansal, Thomas Christensen, S. Nicole Culos-Reed, Colleen A. Cuthbert, Julianna Dreger, Jodi E. Langley, Melanie R. Keats

**Affiliations:** 1 School of Health and Human Performance, Division of Kinesiology, Faculty of Health, Dalhousie University, Halifax, NS B3H 4R2, Canada; al336689@dal.ca (A.F.); jodi.langley@dal.ca (J.E.L.); 2 Physical Activity and Cancer Lab, Dalhousie University and Nova Scotia Health, Halifax, NS B3H 1V7, Canada; thomas.christensen@nshealth.ca; 3 Beatrice Hunter Cancer Research Institute, Halifax, NS B3H 0A2, Canada; 4 Faculty of Kinesiology, University of Calgary, Calgary, AB T2N 1N4, Canada; mannat.bansal@ucalgary.ca (M.B.); nculosre@ucalgary.ca (S.N.C.-R.); jdreger@ucalgary.ca (J.D.); 5 Department of Psychosocial Resources, Arthur J.E. Child Comprehensive Cancer Centre, Alberta Health Services, Calgary, AB T2N 5G2, Canada; 6 Department of Oncology, Cumming School of Medicine, University of Calgary, Calgary, AB T2N 1N4, Canada; cacuthbe@ucalgary.ca; 7 Faculty of Nursing, University of Calgary, Calgary, AB T2N 1N4, Canada

**Keywords:** healthcare provider, exercise, oncology, referral, qualitative research

## Abstract

Exercise is an evidence-based strategy shown to reduce the negative side effects associated with cancer treatment for individuals living with and beyond cancer (LWBC). Healthcare providers (HCPs) play a critical role in promoting exercise for these individuals. Notwithstanding, several barriers hinder HCPs’ ability to discuss and support exercise in clinical practice. EXCEL is an exercise intervention designed to address health disparities in access to exercise oncology resources for rural/remote individuals LWBC, including a referral pathway for HCPs to use. The purpose of this study was to evaluate HCP experiences using the EXCEL exercise referral pathway. We employed an interpretive description methodology, using semi-structured interviews to assess HCP experiences with EXCEL. Overall, HCPs felt empowered to refer to exercise when they were supported in doing so. The findings highlighted (1) a need for a better understanding of the role of exercise professionals and their integration into cancer care; (2) the need for efficient referral systems including embedding referrals into existing health care electronic record systems; and (3) sharing patient feedback with exercise oncology programs back to the HCPs to drive continued referrals.

## 1. Introduction

Two in five Canadians will be diagnosed with cancer each year. Improvements in detection and treatment mean that more and more Canadians are living with and beyond cancer (LWBC) [1]. Individuals LWBC include those from the point of diagnosis, during and post treatment and through the balance of life [2,3]. Individuals LWBC experience many negative side effects both during and after treatment that must be addressed to support their quality of life throughout survivorship.

These negative side effects are multifactorial and include impaired psychosocial (e.g., anxiety, depression) and physical health (e.g., fatigue, peripheral neuropathy), ultimately leading to a reduced quality of life [4,5,6]. One evidence-based way to mitigate negative side effects is exercise [7]. Exercise is a “planned, structured, and repetitive” physical activity that has the “objective of the improvement or maintenance of physical fitness” [8] and mental health [9]. Recent systematic reviews and meta-analyses find that aerobic and resistance exercise increases mental and physical well-being, as well as overall quality of life across several cancer types [10,11,12,13,14]. The American College of Sports Medicine recommends, at a minimum, avoiding physical inactivity and a progression towards 90 min of moderate aerobic activity and two resistance sessions per week [11].

Despite cancer-specific exercise guidelines, individuals LWBC are not moving enough to achieve health benefits [15,16]. Healthcare providers (HCPs; oncologists, oncology nurses, general practitioners, psychologists, rehabilitation professionals, social workers) play a critical role in bringing about exercise behavior change. Explicitly, when HCPs emphasize the importance of exercise to their patients, those patients are more inclined to recognize its significance and are consequently more likely to be active [17,18]. While as many as 90% of HCPs agree that discussing exercise with individuals LWBC is their responsibility [19], fewer than 20% report engaging in exercise discussions and/or refer their patients to an exercise program [20]. Barriers to these discussions/referrals include a lack of time and training, safety concerns, and limited exercise specific knowledge [17,20,21,22,23]. For example, as many as 50% of HCPs do not know what to recommend or how or where to refer their patients to exercise programs [21]. Acknowledging the time and expertise barriers, HCPs are encouraged to simply bring up exercise, and then refer to an exercise specialist who would take on the initiation of a program and monitor the patients’ progress [11,17].

The EXCEL (Exercise for Cancer to Enhance Living well) effectiveness–implementation study aims to improve access to exercise oncology resources for those LWBC in rural and remote regions [24,25]. With a need to foster and support exercise discussions and referrals by HCPs, EXCEL has created and implemented referral pathways. Referral pathways included direct HCP referral (i.e., HCP directly refers their patient to the regional hub), indirect HCP referral (i.e., participant contacts hub after receiving information from an HCP), or self-referral (i.e., participant learns about EXCEL through other sources (e.g., support group, current/former participant). Direct HCP referrals could be made by phone, fax, or email. HCPs were asked to provide a participant name, preferred method of contact (e.g., email, phone), and contact information.

HCPs were provided with in-person and online education to inform them of current exercise guidelines for those LWBC and guidance on how to make referrals to EXCEL. Recruitment posters and brochures were posted in clinical spaces. Information sessions and outreach emails were shared with HCPs every 6 weeks to remind them of the referral process [25]. The purpose of this quality improvement study was to assess the early experiences of HCP with referring to the EXCEL program, including ease of use and satisfaction.

## 2. Materials and Methods

### 2.1. Qualitative Design

This study employed interpretive description (ID), which is a well-established qualitative method that generates knowledge that is specific to clinical practice. Using an inductive approach, ID focuses on exploring and understanding participants’ subjective interpretation of their lived experiences within the broader sociocultural context [26,27]. In this study, ID was used to gain insight into HCPs’ experiences with their use of and satisfaction with an exercise referral pathway within an oncological context. This study’s findings are reported according to the standards of a Consolidated Criteria for Reporting Qualitative Research 32-item checklist [28]. The larger EXCEL study [24] uses the reach, effectiveness, adoption, implementation, and maintenance (RE-AIM) framework for evaluation [29], and the qualitative version of this was used to guide the interviews (RE-AIM Quest) [30]. In addition, the capability, opportunity, motivation, behavior (COM-B) model of behavior change was used to identify HCP competencies, opportunities, and motivations for referral (behavior) [31].

### 2.2. EXCEL Protocol

EXCEL includes a 12-week evidence-based exercise program that combines aerobic and resistance training [24]. It is a Canada-wide study, with referrals made by HCPs to an EXCEL hub site, where a Clinical Exercise Physiologist (CEP) triages and refers out to either in-person or remotely delivered community-based exercise programs run by trained and qualified exercise professionals (QEPs). Participants screened as high risk are triaged to classes supervised by a CEP, while those who are lower risk can participate in classes facilitated by a trained QEP. EXCEL is registered with the clinical trials board (NCT04478851) and has ethics approvals from participating sites.

### 2.3. Setting and Participants

Hub sites led regional outreach activities to build HCP networks and provide general exercise oncology information (i.e., safety, benefits, exercise guidelines, and supportive resources) as well as EXCEL-specific exercise referral information [25]. Interviews were conducted with HCPs (oncologists, oncology nurses, surgeons, physiotherapists, pharmacists, and social workers) from the first two contributing hub sites (Alberta and Nova Scotia) that initiated EXCEL participant recruitment [24]. HCPs were contacted by a site research coordinator by email to request their participation in a quality improvement interview.

### 2.4. Data Collection

Trained interviewers (MB, JL) conducted semi-structured interviews between July 2023 and September 2023 to gain a deeper understanding of HCP perspectives and experiences using the EXCEL referral pathway. In brief, HCPs were asked to comment on their beliefs, values, and experiences with physical activity and exercise in general as well as their perceptions and potential value of physical activity and exercise in supportive cancer care. HCPs were also asked to share their experiences with initiating and supporting patient-initiated exercise discussions and if they provided their patients with additional information about exercise and EXCEL, including what sources of information were used (e.g., poster, brochure). Barriers to exercise discussions, sharing of information, and perceived value of the CEP in facilitating exercise referral were also explored. Repeat interviews were not conducted and transcripts were not returned to participants for additional comments.

These data represent the findings of the first quality improvement cycle conducted with participants from the initial two hub sites (Fall 2020 and Winter 2021) [25]. The semi-structured interview guide (Supplementary File) addressed HCP general perceptions of and experiences with exercise programs in cancer care, barriers and facilitators to exercise discussion and referrals in cancer care, experiences with the EXCEL exercise referral pathway, and perceptions of the supporting role of CEPs in cancer care.

Interviews were conducted in person and online using Zoom videoconferencing based on participant preference. Interviews were audio-recorded and transcribed verbatim. No others were present during the interviews.

### 2.5. Data Analysis

Transcribed interviews were imported into NVivo software version 14 [32] which was then used to organize and code the transcripts. The first author (AF) coded each interview by first familiarizing herself with the data by reading and re-reading each transcript, making written comments, following up with interviewers for additional context, and writing short summaries describing the main concepts shared by participants. Consistent with the principles of ID, we used thematic analysis to guide the development of codes and themes [33]. A codebook outlining codes, their definitions, and examples was developed. Codes were then refined through iterative rounds of discussion and reviewing transcripts and coding with interviewers (JL, MB) and co-authors (NCR, CC, MK). Representative quotations from emerging themes are presented.

We acknowledge that the personal biases, training, and experiences of our research team can influence our perspectives and interpretation of participant responses. To address the significant impact of researcher positionality and enhance rigor, the first author (AF) employed reflexive practices (e.g., critical reflection, researcher position) and engaged in critical discussions with co-authors (MB, JL, NCR, CC, MK) through the analytical process.

AF, a cisgender female, was a Masters of Science in Kinesiology student and volunteer in an exercise oncology research lab at the time of this study. While reviewing and interpretating the interviews, AF acknowledged a personal bias favoring the benefits of exercise for her own personal well-being as well as those LWBC. While reviewing and interpreting the interviews, AF made a conscious effort to reflect on her own biases and assumptions and engaged in ongoing self-reflection to foster a more authentic understanding of the participants’ experiences. She did not have any previous relationships with any of the participants. By maintaining transparency in her role, we aim to improve the credibility and trustworthiness of the research process.

## 3. Results

A total of 26 HCPs from the NS hub and 20 HCPs from the AB hub were invited to participate in an interview. Thirteen HCP interviews were conducted (6 from NS and 7 from the AB hub), with each interview lasting between 17 and 45 min. All participants had previously referred patients to EXCEL and regarded exercise as being critical to the overall well-being and empowerment of individuals LWBC. Participant characteristics are presented in Table 1.

### 3.1. Achieving More by Doing Less

The overarching narrative shared by HCPs was that streamlining referral and optimizing resources (i.e., access to trained exercise professionals and safe, evidence-based exercise oncology programming) promotes an increase in their referrals to exercise oncology programming by ultimately being asked to do less. This narrative was supported by the emergence of three underlying subthemes and corresponding facilitators (Table 2).

#### 3.1.1. Sub-Theme 1: Optimizing the Role of CEPs in Multidisciplinary Cancer Care

The first theme characterizes exercise as a “missing piece” in healthcare and underscores the critical need for the integration of an exercise specialist within clinical cancer care to support exercise discussions and facilitate exercise referrals.

“*100% I think [CEPs have] always been a missing piece. I think a PT [physiotherapist] and a CEP is a perfect team in combination with our other rehab people [for] both our acute and certainly our chronic health care populations. It really is a missing piece in health care.*”

Allied HCP

“*[They/CEPs] bring a better depth of knowledge to what I can tell them.*”

Allied HCP

As illustrated in the following quotes, although many HCPs were well-versed on the role of the CEP, some were less sure or were unable to distinguish their role from that of a physiotherapist.

“*I don’t know if I really understand the nuances … between a physiotherapist and an exercise physiologist.*”

Allied HCP

“*So, when I describe it to my patients, I say it’s not like the gym guy that’s going to yell at you to do push-ups, it’s someone that has, you know, a very medical…a medical and an understanding of human anatomy. And from that perspective, understanding of like movement, like the signs of movement, kinesiology.*”

Allied HCP

With an incomplete understanding of the complementary and supporting role of the CEP within cancer care, some suggested that additional details regarding CEP training and scope of practice would be useful for them to understand.

“*…knowledge of…the role of the CEP and what their background is and what they can offer and how they can work collaboratively with in the team together with the rehab professionals you know specifically but that bigger team as well…education on both those facets [is needed].*”

Allied HCP

While the findings highlight a meaningful knowledge gap surrounding the expertise and role of CEPs among some HCPs, overall, there was an appreciation of their expertise and support for the integration into cancer care.

#### 3.1.2. Theme 2: Simplicity Drives Sustainability

Recognizing and voicing their highly time-constrained clinical settings, HCPs emphasized the need for streamlined and time-efficient referral systems.

“*…there’s so many things to do with a visit, and there’s so much ground to cover, and you’re getting pages about other patients, and you’re running behind in the clinic, that it’s so difficult to give the focus to everything that it requires.*”

Physician

“*… when [my patient and I are] talking…I do bring [exercise] up, and it is important, but it’s not something that I can address at that very moment.*”

Nurse

Some HCPs perceived the current EXCEL referral system to be a low burden due to strong communication, an efficient referral pathway, a trusted referral source, and low paperwork burden.

“*I think we’ve um really established a good system and have had a lot of communication around that.*”

Allied HCP

“*Yeah. Like the smaller the amount of paperwork, the better. If I can just give you guys their information, that really helps. It makes me more likely to do it…I usually like that I can put it to somebody else to follow up on.*”

Nurse

Notwithstanding the largely favorable view of the EXCEL referral pathway, some suggested that embedding referrals into current medical electronic systems would be an important addition for integrating referrals more seamlessly into clinical practice.

“*I think maybe the easier thing would be if you were a bookable in [name of booking system]…*”

Allied HCP

While EXCEL has provided a feasible and easy-to-use system for many HCPs, referrals may be further simplified by integrating referrals into medical record systems.

#### 3.1.3. Theme 3: Generating a Positive Feedback Loop

The final theme highlights how a positive patient endorsement of EXCEL/exercise experiences is noticed and appreciated by HCPs. This endorsement was found to be a potential positive driver among HCPs for continued referral to exercise programming.

“*I wonder if there’s like patient testimonials that would help the clinicians. Because I know for me, if I’m starting to refer either like somebody new or a different thing, and the patient comes back after and is like, ‘Oh, my God, I feel so great. This was… Thank you for doing that,’ that just like builds that habit.*”

Allied HCP

“*…the patients who I have had who have engaged with your programs have finished and said, ‘I wish there wasn’t an end to this. I’d love to keep going. And I feel so much better.’*”

Allied HCP

Overall, HCPs appreciate and identify positive patient feedback and improved health outcomes as motivation to continue referring patients to exercise programming.

## 4. Discussion

Overall, consistent with the work of others, HCPs in the present study expressed positive attitudes and support for exercise as an important supportive therapy in cancer care [34]. Guided by the RE-AIM Quest framework [30], the findings from this qualitative study revealed that HCPs who have referred to EXCEL perceive exercise as a critical component and “missing piece” in supportive cancer care (Reach). Notwithstanding, it is evident that the majority of HCPs are not routinely engaging in exercise discussions or referring cancer patients to tailored exercise programs [20,35]. Recognizing the highly time-constrained clinical setting [35,36], HCPs valued the streamlined EXCEL referral pathway (Adoption, Implementation), but would like to see further integration of the referral process into existing electronic medical systems. Notably, the use of electronic referrals in related clinical settings has shown to increase the number of referrals, improve access to specialized care, and facilitate patient–provider communication and the coordination and integration of health care services [37,38,39,40,41,42].

In addition to the need for an efficient referral pathway, HCPs also acknowledged the critical role of EXCEL CEPs in facilitating exercise discussions and referrals to exercise programs (Implementation, Effectiveness). Others have similarly highlighted the need for the integration of qualified exercise professionals specializing in cancer care into the clinical team [18,21,36,43,44]. Doing so would free the HCP from needing to engage in lengthy exercise counselling sessions that they do not have the confidence, expertise, and/or time to conduct [21,36,45,46]. Notwithstanding, the findings of the present study suggest that there remains a meaningful knowledge gap with respect to the CEPs (or equivalent) scope of practice and role within cancer care for some HCPs (Adoption, Implementation). Mizrahi and colleagues similarly found that while the majority of oncology professionals value the supporting role of exercise professionals, as many as one-third of HCPs do not understand the function of exercise professionals within cancer care [47]. A lack of understanding of the functions of the CEP and other trained qualified exercise professionals may serve to hinder HCP referrals, as HCPs need to trust in the exercise professionals’ ability to provide the appropriate care for their patients [48]. Several studies have highlighted the need for ongoing HCP education regarding the empirical value and safety of exercise as well as how to initiate and support exercise discussions and/or referrals for those LWBC [17,48]. However, few studies have identified the need for providing a better understanding of the role of exercise professionals within the multidisciplinary cancer care team.

Finally, positive patient feedback was expressed by the HCPs in this study as an important factor influencing their motivation to continue to endorse and refer patients to exercise programming (Maintenance). Ezenwankwo et al. [49] similarly reported that patient endorsement/positive feedback was an important motivator for HCPs to continue referring patients to exercise programs. Given that a lack of exercise knowledge and a need for continued HCP education has been widely reported [34] and the large body of literature showing that a lack of HCP awareness of the benefits of exercise for those LWBC is a barrier to exercise referral [17,36,46], providing HCPs with patient outcome data and/or patient experience stories may be a useful strategy to reinforce their decision to refer patients to exercise programming [50]. Ongoing work at the EXCEL primary site (Calgary, AB) includes an HCP feedback form to facilitate better communication and build awareness within clinical teams on the patient experience with exercise.

### Limitations

While the study provides rich data to inform the continued implementation of referral pathways in EXCEL, HCPs who responded to our invitation had all referred to EXCEL. As EXCEL has expanded across Canada, future work will continue to engage and gather data from HCPs from other participating provinces (British Columbia, Ontario, Quebec, Prince Edward Island, and Newfoundland and Labrador), and efforts will be made to gather input from HCPs who have not referred to EXCEL or who express ongoing concerns with referring patients to exercise in general. Continuing to address barriers and build systems that support the role of HCPs in referral is critical for the implementation of exercise in standard cancer care.

## 5. Conclusions

HCPs feel empowered to refer more patients to exercise when they have access to an efficient referral pathway, know they are referring to trained exercise professionals, and receive positive patient feedback about their program experience. EXCEL and provincial initiatives [39,51] continue to build and advocate for exercise within supportive cancer care to ultimately enhance the quality of life of individuals LWBC.

## Figures and Tables

**Table 1:** Participant characteristics.

	N
Region	
Alberta Hub (20 invited)	
Alberta	4
Saskatchewan	2
Manitoba	1
Northwest Territories	0
Nova Scotia Hub (26 invited)	6
Profession	
Oncologist	1
Surgeon	1
General practitioner	2
Nurse	3
Social worker	2
Physiotherapist	1
Pharmacist	1
Not reported	2
Years of Clinical Experience (Range)	7–25

**Table 2:** Healthcare provider experiences on referring those living with and beyond cancer to EXCEL ^1^.

Sub-Theme	Facilitator
Optimizing the role of CEPs ^2^ in multidisciplinary cancer care	Knowledge and education on role of CEPs
Simplicity drives sustainability	Integration of referrals with existing medical record systems
Generating a positive feedback loop	Patient testimonials from their EXCEL experience

^1^ EXCEL = Exercise for Cancer to Enhance Living well, ^2^ CEP = certified exercise professional.

## Data Availability

Data are unavailable due to privacy restrictions.

## Supplementary Materials

The following supporting information can be downloaded at: https://www.mdpi.com/article/10.3390/curroncol32030181/s1, File S1: COREQ Checklist; File S2: EXCEL HCP Consent Form; File S3: HCP Interview Guide
