# Supplementary material for: Exploring Healthcare Provider Experiences with the EXCEL Exercise Referral Pathway for Individuals Living with and Beyond Cancer
Source: Curr Oncol. 2025 Mar 20;32(3):181. doi: 10.3390/curroncol32030181 (PMC11941420; doi:10.3390/curroncol32030181)
Supplement: Supplementary file 1 [file curroncol-32-00181-s001.zip › EXCEL-HCP-Consent-Form-merged.pdf]

Informed Consent Form for Participation in a Research Study

EXCEL: EXercise for Cancer to Enhance Living well Study

(A study to evaluate the benefit of a community-based exercise program for cancer survivors in rural and remote Canada)

Clinical Trial Resgistry NCT04478851

Principal Investigator: Dr. Melanie Keats, PhD
School of Health and Human Performance, Dalhousie University
And NSHA Affiliated Scientist, Division of Medical Oncology

Sponsor/Funder(s): The Canadian Institutes of Health Research/ Canadian Cancer Society and the Alberta Cancer Foundation

You are being invited to participate in a research study because you are a healthcare provider, involved in the care of those living with and beyond cancer. This consent form provides detailed information about the study to assist you with making an informed decision. Please read this document carefully and ask any questions you may have. All questions should be answered to your satisfaction before you decide whether to participate.

The study staff will tell you about timelines for making your decision. You may find it helpful to discuss the study with family and friends so that you can make the best possible decision within the given timelines.

Taking part in this study is voluntary. You may choose not to take part or, if you choose to participate, you may leave the study at any time without giving a reason. Deciding not to take part or deciding to leave the study will not result in any penalty.

The principal investigator, who is one of the researchers, the site research coordinator, or research assistant, will discuss this study with you and will answer any questions you may have. If you do consent to participate in this study, you will need to sign and date this consent form. You will receive a copy of the signed form.

WHAT IS THE BACKGROUND INFORMATION FOR THIS STUDY?

The growing population of cancer survivors in Canada has brought attention to the long term toll of cancer and its treatment on the body, mind, and overall health of survivors. Exercise is an effective intervention that can optimize the health and well-being of cancer survivors and possibly reduce rates of cancer recurrence and secondary cancers.

The Nova Scotia Health Research Ethics Board, which oversees the ethical acceptability of research involving humans, has reviewed and granted ethics approval for this study.

WHY IS THIS STUDY BEING DONE?

The purpose of this study is to evaluate the benefit of a community-based or online exercise program for cancer survivors who live in rural and remote locations. The study is called EXCEL and includes an evidence-based exercise Our aim is to provide an exercise program to cancer survivors living in rural and remote locations to promote adoption of an active lifestyle in order and improve health outcomes. EXCEL will increase accessibility to exercise as a supportive cancer care resources for all cancer survivors. As part of our quality improvement cycles, we are interested in understanding your experiences as a healthcare provider with EXCEL.

HOW MANY PEOPLE WILL TAKE PART IN THIS STUDY?

Between 10-15 healthcare providers and 10-15 fitness professionals across Canada will take part in these interviews. Of those, 5 from each group will be from Nova Scotia.

WHAT WILL HAPPEN DURING THIS STUDY?

If you agree to participate, the interview will be one-on-one with the research coordinator or research assistant, and will take approximately 20-25 minutes to complete.The purpose of these interviews is to get your feedback on your experience with EXCEL, specifically surrounding the logistics of referring your patients to EXCEL.

WHAT ARE THE POTENTIAL SIDE EFFECTS FROM PARTICIPATING IN THIS STUDY?

You will be responding to questions regarding your experience with the study. You may refuse to answer any questions that you do not feel comfortable with.

WHAT ARE THE BENEFITS OF PARTICIPATING IN THIS STUDY?

Participation in this study may or may not be of personal benefit to you. However, based on the results of this study, it is hoped that we can learn how to better support clinical referral of patients into community based exercise oncology programs.

WHAT ARE MY RESPONSIBILITIES AS A STUDY PARTICIPANT?

If you choose to participate in this study, you will be asked to particiate in one interview, which will require a 20-25 minute time period. The research coordinator or research assistant will ask you a series of questions related to your experience with the referral process in EXCEL.

CAN I CHOOSE TO LEAVE THIS STUDY EARLY?

You can choose to end your participation in this research (called early withdrawal) at any time without having to provide a reason. If you choose to withdraw early from the study without finishing the interview, you are encouraged to contact the principal investigator or research coordinator.

You may withdraw your permission to use information that was collected about you for this study at any time by letting the research coordinator know. However, this would also mean that you withdraw from the study. Information that was recorded before you withdrew will be used by the researchers for the purposes of the study, but no additional information will be collected or sent to the sponsor after you withdraw your permission.

HOW WILL MY PERSONAL INFORMATION BE KEPT CONFIDENTIAL?

If you decide to participate in this study, the principal investigator and study staff will only collect the information they need for this study.

Records identifying you will be kept confidential to the extent permitted by the applicable laws, will not be disclosed or made publicly available, except as described in this consent document.

Authorized representatives of the following organization may look at your identifiable study records at the site where these records are held for quality assurance purposes and/or to verify that the information collected for the study is correct and follows proper laws and guidelines:

- The NSHA REB and people working for or with the NSHA REB because they oversee the ethical conduct of research studies within the NSHA;

All information collected during this study will be kept confidential and will not be shared with anyone outside the study unless required by law. You will not be named in any reports, publications, or presentations that may come from this study.

Authorized representatives of the above organization may **receive** information related to the study that will be kept confidential in a secure online server at NS Health used in current or future relevant health research. Your name or other information that may identify you will not be provided (i.e., the information will be de-identified). The records received by these organizations will be coded with a number. The key that indicates what number you have been assigned will be kept secure by the researchers directly involved with your study and will not be released. To protect your identity, the information from the interview will only include your study ID. Collected information during the interview about your position (role, years in cancer care) will be removed from transcription and not be included within the qualitative data analysis.

If the results of this study are published, your identity will remain confidential. It is expected that the information collected during the study will be used in analyses and will be published and/or presented to the scientific community at meetings and in journals, but your identity will remain confidential. It is expected that the study results will be published as soon as possible after completion. This information may also be used as part of a submission to regulatory authorities around the world to support the approval of this intervention.

Even though the likelihood that someone may identify you from the study data is very small, it can never be completely eliminated. Every effort will be made to keep your identifiable information confidential, and to follow the ethical and legal rules about collecting, using and disclosing this information.

WILL I BE COMPENSATED FOR PARTICIPATING IN THIS STUDY?

Although no funds have been set aside to compensate you in the event of injury or illness related to the study procedures, you do not give up any of your legal rights for compensation by signing this form.

WHAT ARE MY RIGHTS AS A PARTICIPANT IN THIS STUDY?

You will be told, in a timely manner, about new information that may be relevant to your willingness to stay in this study. You have the right to be informed of the results of this study once the entire study is complete. If you would like to be informed of these results, please contact the principal investigator.

The results of this study will be available on a clinical registry; refer to the section titled "Where can I find online information about this study?". Your rights to privacy are legally protected by federal and provincial laws that require safeguards to ensure that your privacy is respected.

By signing this form you do not give up any of your legal rights against the hospital, investigators, sponsor, involved institutions for compensation or their agents, nor does this form relieve these parties from their legal and professional responsibilities.

IS THERE CONFLICT OF INTEREST RELATED TO THIS STUDY?

There are no conflicts of interest declared between the principal investigator and sponsor of this study.

WHERE CAN I FIND ONLINE INFORMATION ABOUT THIS STUDY?

A description of this clinical trial will be available on http://www.clinicaltrials.gov, as required by U.S. Law. This Web site will not include information that can identify you. At most, the Web site will include a summary of the results. You can search this Web site at any time.

The study registration number to use this website is: NCT04478851

WHO DO I CONTACT FOR QUESTIONS?

If you have questions about taking part in this study, or if you suffer a research-related injury, you should talk to the research coordinatior or principal investigator. These person(s) are :

Ms. Jodi Langley (Research Coordinator)	Ph: 902-473-2035<br>Email: jodi.langley@nshealth.ca
Dr. Melanie Keats, PhD (Principal Investigator)	Ph: 902-494-7173<br>Email: melanie.keats@nshealth.ca

You have the right to all information that could help you make a decision about participating in this study. You also have the right to ask questions about this study and your rights as a research participant, and to have them answered to your satisfaction before you make any decision. You also have the right to ask questions and to receive answers throughout this study. You have the right to withdraw your consent at any time.

If you have questions about your rights as a research participant, and/or concerns or complaints about this research study, you can contact the Nova Scotia Health Authority Research Ethics Board manager at 902-473-8426 or Patient Relations at (902) 473-2133 or 1-855-799-0990 or healthcareexperience@nshealth.ca.

VERBAL CONSENT

Verbal consent will be given prior to collecting any data.

Informed Consent Form for Participation in a Research Study

EXCEL: EXercise for Cancer to Enhance Living well Study

(A study to evaluate the benefit of a community-based exercise program for cancer survivors in rural and remote Canada)

Protocol ID: *HREBA.CC-20-0098*

Principal Investigator: Dr. Nicole Culos-Reed, PhD
Health & Wellness Lab, Faculty of Kinesiology
University of Calgary
Phone: 403-220-7540

Sponsor/Funder(s): The Canadian Institutes of Health Research/ Canadian Cancer Society and the Alberta Cancer Foundation

You are being invited to participate in a research study because you are a healthcare provider, involved in the care of those living with and beyond cancer. This consent form provides detailed information about the study to assist you with making an informed decision. Please read this document carefully and ask any questions you may have. All questions should be answered to your satisfaction before you decide whether to participate.

The study staff will tell you about timelines for making your decision. You may find it helpful to discuss the study with family and friends so that you can make the best possible decision within the given timelines.

Taking part in this study is voluntary. You may choose not to take part or, if you choose to participate, you may leave the study at any time without giving a reason. Deciding not to take part or deciding to leave the study will not result in any penalty.

The principal investigator, who is one of the researchers, the site research coordinator, or research assistant, will discuss this study with you and will answer any questions you may have. If you do consent to participate in this study, you will need to sign and date this consent form. You will receive a copy of the signed form.

WHAT IS THE BACKGROUND INFORMATION FOR THIS STUDY?

The growing population of cancer survivors in Canada has brought attention to the long term toll of cancer and its treatment on the body, mind, and overall health of survivors. Exercise is an effective intervention that can optimize the health and well-being of cancer survivors and possibly reduce rates of cancer recurrence and secondary cancers.

The Health Research Ethics Board of Alberta – Cancer Committee (HREBA-CC), which oversees the ethical acceptability of research involving humans, has reviewed and granted ethics approval for this study.

WHY IS THIS STUDY BEING DONE?

The purpose of this study is to evaluate the benefit of a community-based or online exercise program for cancer survivors who live in rural and remote locations. The study is called EXCEL and includes an evidence-based exercise program (Alberta Cancer Exercise, ACE; Ethics ID: HREBA-CC-16-0905). ACE has been successfully implemented in urban centres throughout Alberta. Our aim is to provide an exercise program to cancer survivors living in rural and remote locations to promote adoption of an active lifestyle in order and improve health outcomes. EXCEL will increase accessibility to exercise as a supportive cancer care resources for all cancer survivors. As part of our quality improvement cycles, we are interested in understanding your experiences as a healthcare provider with EXCEL.

HOW MANY PEOPLE WILL TAKE PART IN THIS STUDY?

Between 10 and 15 healthcare providers across Canada will take part in these interviews.

WHAT WILL HAPPEN DURING THIS STUDY?

If you agree to participate, the interview will be one-on-one with the research coordinator or research assistant, and will take approximately 15-20 minutes to complete.The purpose of these interviews is to get your feedback on your experience with EXCEL, specifically surrounding the logistics of referring your patients to EXCEL.

WHAT ARE THE POTENTIAL SIDE EFFECTS FROM PARTICIPATING IN THIS STUDY?

You will be responding to questions regarding your experience with the study. You may refuse to answer any questions that you do not feel comfortable with.

WHAT ARE THE BENEFITS OF PARTICIPATING IN THIS STUDY?

Participation in this study may or may not be of personal benefit to you. However, based on the results of this study, it is hoped that we can learn how to better support clinical referral of patients into community based exercise oncology programs.

WHAT ARE MY RESPONSIBILITIES AS A STUDY PARTICIPANT?

If you choose to participate in this study, you will be asked to particiate in one interview, which will require a 15-20 minute time period. The research coordinator or research assistant will ask you a series of questions related to your experience with the referral process in EXCEL.

CAN I CHOOSE TO LEAVE THIS STUDY EARLY?

You can choose to end your participation in this research (called early withdrawal) at any time without having to provide a reason. If you choose to withdraw early from the study without finishing the interview, you are encouraged to contact the principal investigator or research coordinator.

You may withdraw your permission to use information that was collected about you for this study at any time by letting the research coordinator know. However, this would also mean that you withdraw from the study. Information that was recorded before you withdrew will be used by the researchers for the purposes of the study, but no additional information will be collected or sent to the sponsor after you withdraw your permission.

HOW WILL MY PERSONAL INFORMATION BE KEPT CONFIDENTIAL?

If you decide to participate in this study, the principal investigator and study staff will only collect the information they need for this study.

Records identifying you will be kept confidential to the extent permitted by the applicable laws, will not be disclosed or made publicly available, except as described in this consent document.

Authorized representatives of the following organization may look at your identifiable study records at the site where these records are held for quality assurance purposes and/or to verify that the information collected for the study is correct and follows proper laws and guidelines:

- The Health Research Ethics Board of Alberta – Cancer Committee, which oversees the ethical conduct of this study

All information collected during this study will be kept confidential and will not be shared with anyone outside the study unless required by law. You will not be named in any reports, publications, or presentations that may come from this study.

Authorized representatives of the above organization may **receive** information related to the study that will be kept confidential in a secure online server, under Dr. Culos-Reed in the Faculty of Kinesiology at the University of Calgary, and may be used in current or future relevant health research. Your name or other information that may identify you will not be provided (i.e., the information will be de-identified). The records received by these organizations will be coded with a number. The key that indicates what number you have been assigned will be kept secure by the researchers directly involved with your study and will not be released. To protect your identity, the information from the interview will only include your study ID. Collected information during the interview about your position (role, years in cancer care) will be removed from transcription and not be included within the qualitative data analysis.

If the results of this study are published, your identity will remain confidential. It is expected that the information collected during the study will be used in analyses and will be published and/or presented to the scientific community at meetings and in journals, but your identity will remain confidential. It is expected that the study results will be published as soon as possible after completion. This information may also be used as part of a submission to regulatory authorities around the world to support the approval of this intervention.

Even though the likelihood that someone may identify you from the study data is very small, it can never be completely eliminated. Every effort will be made to keep your identifiable information confidential, and to follow the ethical and legal rules about collecting, using and

disclosing this information.

WILL I BE COMPENSATED FOR PARTICIPATING IN THIS STUDY?

Although no funds have been set aside to compensate you in the event of injury or illness related to the study procedures, you do not give up any of your legal rights for compensation by signing this form.

WHAT ARE MY RIGHTS AS A PARTICIPANT IN THIS STUDY?

You will be told, in a timely manner, about new information that may be relevant to your willingness to stay in this study. You have the right to be informed of the results of this study once the entire study is complete. If you would like to be informed of these results, please contact the principal investigator.

The results of this study will be available on a clinical registry; refer to the section titled "Where can I find online information about this study?". Your rights to privacy are legally protected by federal and provincial laws that require safeguards to ensure that your privacy is respected.

By signing this form you do not give up any of your legal rights against the hospital, investigators, sponsor, involved institutions for compensation or their agents, nor does this form relieve these parties from their legal and professional responsibilities.

IS THERE CONFLICT OF INTEREST RELATED TO THIS STUDY?

There are no conflicts of interest declared between the principal investigator and sponsor of this study.

WHERE CAN I FIND ONLINE INFORMATION ABOUT THIS STUDY?

A description of this clinical trial will be available on http://www.clinicaltrials.gov, as required by U.S. Law. This Web site will not include information that can identify you. At most, the Web site will include a summary of the results. You can search this Web site at any time.

The study registration number to use this website is: NCT04478851

WHO DO I CONTACT FOR QUESTIONS?

If you have questions about taking part in this study, or if you suffer a research-related injury, you should talk to the research coordinatior or principal investigator. These person(s) are :

Ms. Julianna Dreger CSEP-CEP (Research Coordinator)	Ph: 403-210-8482<br>Email: jdreger@ucalgary.ca
Dr. Nicole Culos-Reed, PhD (Principal Investigator)	Ph: 403-220-7540<br>Email: nculosre@ucalgary.ca

If you have questions about your rights as a participant or about ethical issues related to this study and you would like to talk to someone who is not involved in the conduct of the study, please contact the Office of the Health Research Ethics Board of Alberta – Cancer Committee at:

Telephone: 780-423-5727　　Toll Free: 1-877-423-5727

SIGNATURES

Part 1 - to be completed by the potential participant.

	**Yes**	**No**
Do you understand that you have been asked to take part in a research study?	☐	☐
Do you understand why this study is being done?	☐	☐
Do you understand the potential benefits of taking part in this study?	☐	☐
Do you understand the risks of taking part in this study?	☐	☐
Do you understand what you will be asked to do should you decide to take part in this study?	☐	☐
Do you understand the alternatives to participating in this study?	☐	☐
Do you understand that you are free to leave the study at any time, without out having to give reason?	☐	☐
Do you understand who will see your records, including those that identify you?	☐	☐
Do you understand that by signing this consent form that you do not give up any of your legal rights?	☐	☐
Have you had enough opportunity to ask questions and discuss this study?	☐	☐

By signing this form I agree to participate in this study.

Signature of Participant	PRINTED NAME	Date

Part 2 - to be completed by the principal investigator or designee who conducted the informed consent discussion. Only compete this section if the potential participant has **agreed** to participate.

I believe that the person signing this form understands what is involved in the study and has freely decided to participate.

______________________	______________________	______________
Signature of Person Conducting the Consent Discussion	PRINTED NAME	Date

You will be given a copy of this signed and dated consent form prior to participating in this study.
