# Supplementary material for: Exploring Healthcare Provider Experiences with the EXCEL Exercise Referral Pathway for Individuals Living with and Beyond Cancer
Source: Curr Oncol. 2025 Mar 20;32(3):181. doi: 10.3390/curroncol32030181 (PMC11941420; doi:10.3390/curroncol32030181)
Supplement: Supplementary file 1 [file curroncol-32-00181-s001.zip › HCP Interview Guide.pdf]

Semi-structured interview questions
EXCEL HCPs

Background questions
- What are your views on PA/exercise? Are you physically active yourself?
- Do you refer to other wellness/exercise/PA resources? If so, what?

First, I'd like to ask some background questions about yourself:

1. Please tell me about your role in cancer care/healthcare, how many years you've been in your role, and your involvement with the EXCEL study. If you're comfortable, please share your pronouns and biological sex.

Next, I'd like to ask you about your experience with the EXCEL study:

1. What are your perceptions of physical activity or exercise in general, or EXCEL if you are familiar with it, for individuals living with and beyond cancer?

2. What is it's use / value / overall role in cancer care? How do you see it supporting those going through cancer treatment? Into survivorship?

I'd now like to discuss your experiences with discussing EXCEL in your clinical setting:

1. Did you have any patients bring it up to you (vs you to them)?

2. Are you providing information about the EXCEL study to potential participants?
   a. If no – go to 2 and probe on 'what barriers do you have to exercise oncology/EXCEL discussions/info being presented
   b. Tell me about your experiences with providing resources.
      i. Probe: What do you typically use? Study brochure, posters, etc. do you have enough info??

3. We know there are many barriers in within clinical settings that make it hard to discuss exercise. Can you tell us what barriers you feel make it hard for you to introduce EXCEL to your patients?

4. Beyond simply informing about EXCEL, do you have conversations or provide resources to your patients about exercise benefits for individuals with cancer?
   a. If 'yes', what benefits do you discuss? Do you find patients receptive to this type of conversation?
   b. If 'no', why not? How might EXCEL support you in being able to facilitate these types of conversations?

5. Are you familiar with a CEP (say full name)?
   a. Depending on yes/no response, have discussion about what they think value of CEP within cancer care is.
      i. Probe: In busy clinic environments, a CEP may be a key HCP to facilitate patients learning more about exercise, being screened, and being referred into exercise programs.
         1. What do you think about this?
         2. Thoughts about barriers to doing this?

3. How it might be helpful in your role in supporting patients to live well?

Lastly, I’d like to ask you about continued participation and communication with the EXCEL study:

1. Do you plan to continue to refer to the EXCEL study in the future?
   a. If ‘yes’, can the EXCEL team provide any additional resources for support?
   b. If ‘no’, what can the EXCEL team provide to might help with your potential referrals to EXCEL?

2. Our communication involves email reminders for EXCEL referrals, study newsletters that provide updates on current progress, and direct outreach via phone call. Would you like to continue to receive this type of communication? Do you have a preference or suggestions for future communication from the EXCEL study team?

This is the end of the interview. Thank you for participating in the interview and the study in general. Don’t hesitate to contact me in case you have any questions or concerns.
